# Patterns of SARS-CoV-2-specific humoral and cellular immune response in actively treated patients with solid cancer following prime BNT162b2 COVID-19 vaccination: results from phase IV CoVigi trial

**DOI:** 10.1177/17588359251316224

**Published:** 2025-05-17

**Authors:** Radka Lordick Obermannova, Iveta Selingerova, Regina Demlova, Dominika Okruhlicova, Jiri Nevrlka, Katerina Cerna-Pilatova, Kristina Greplova, Zdenka Cermakova, Dalibor Valik, Igor Kiss, Marketa Palacova, Alexandr Poprach, Hana Lejdarova, Sarka Selvekerova, Martina Vaneckova, Lenka Zdrazilova-Dubska

**Affiliations:** Department of Comprehensive Cancer Care, Masaryk Memorial Cancer Institute, Zluty kopec 7, Brno 65653, Czech Republic; Department of Comprehensive Cancer Care, Faculty of Medicine, Masaryk University, Brno, Czech Republic; Department of Pharmacology and CREATIC, Faculty of Medicine, Masaryk University, Brno, Czech Republic; Department of Pharmacology and CREATIC, Faculty of Medicine, Masaryk University, Brno, Czech Republic; Department of Mathematics and Statistics, Faculty of Science, Masaryk University, Brno, Czech Republic; Department of Laboratory Medicine, Masaryk Memorial Cancer Institute, Brno, Czech Republic; Department of Pharmacology and CREATIC, Faculty of Medicine, Masaryk University, Brno, Czech Republic; Clinical Trials Unit, Masaryk Memorial Cancer Institute, Brno, Czech Republic; Department of Laboratory Medicine, University Hospital Brno, and Department of Laboratory Methods, Faculty of Medicine, Masaryk University, Brno, Czech Republic; Department of Pharmacology and CREATIC, Faculty of Medicine, Masaryk University, Brno, Czech Republic; Department of Laboratory Medicine, University Hospital Brno, and Department of Laboratory Methods, Faculty of Medicine, Masaryk University, Brno, Czech Republic; Department of Pharmacology and CREATIC, Faculty of Medicine, Masaryk University, Brno, Czech Republic; Department of Laboratory Medicine, University Hospital Brno, and Department of Laboratory Methods, Faculty of Medicine, Masaryk University, Brno, Czech Republic; Department of Laboratory Medicine, Masaryk Memorial Cancer Institute, Brno, Czech Republic; Department of Laboratory Medicine, Masaryk Memorial Cancer Institute, Brno, Czech Republic; Department of Pharmacology and CREATIC, Faculty of Medicine, Masaryk University, Brno, Czech Republic; Department of Laboratory Medicine, University Hospital Brno, and Department of Laboratory Methods, Faculty of Medicine, Masaryk University, Brno, Czech Republic; Department of Comprehensive Cancer Care, Masaryk Memorial Cancer Institute, Brno, Czech Republic; Department of Comprehensive Cancer Care, Faculty of Medicine, Masaryk University, Brno, Czech Republic; Department of Comprehensive Cancer Care, Masaryk Memorial Cancer Institute, Brno, Czech Republic; Department of Comprehensive Cancer Care, Masaryk Memorial Cancer Institute, Brno, Czech Republic; Department of Comprehensive Cancer Care, Faculty of Medicine, Masaryk University, Brno, Czech Republic; Transfusion and Tissue Department, University Hospital Brno, Brno, Czech Republic; Clinical Trials Unit, Masaryk Memorial Cancer Institute, Brno, Czech Republic; Clinical Trials Unit, Masaryk Memorial Cancer Institute, Brno, Czech Republic; Department of Pharmacology and CREATIC, Faculty of Medicine, Masaryk University, Brno, Czech Republic; Department of Laboratory Medicine, University Hospital Brno, and Department of Laboratory Methods, Faculty of Medicine, Masaryk University, Brno, Czech Republic

**Keywords:** COVID-19 vaccination, immune response, SARS-CoV-2 antibodies, SARS-CoV-2-specific T-cell response, solid cancer

## Abstract

**Background:**

Cancer patients are particularly vulnerable during the COVID-19 pandemic. Vaccinations are essential in controlling the pandemic. However, due to their exclusion from clinical trials for COVID-19 vaccines, there is limited data on the vaccines’ effectiveness and safety for this group.

**Objectives:**

We evaluated humoral (anti-S antibody) and cellular (T-cell) immune response in patients with solid cancer on systemic anticancer treatment versus healthy controls prime-vaccinated by the BNT162b2 COVID-19 mRNA vaccine.

**Methods:**

CoVigi was the phase IV prospective open-label non-randomized multicentric clinical trial evaluating anti-S and anti-N SARS-CoV-2 antibodies and SARS-CoV-2-specific T-cell response by IFN-γ-release assay in several time points during the prime COVID-19 mRNA vaccination (prior to the first vaccine dose, prior to the second dose, at 4–8 weeks, at 3 months, and 6 months after vaccination). Immune response was analyzed in the context of previous SARS-CoV-2 infection and anticancer therapy (chemotherapy (CT) + monoclonal antibodies (mAb), mAb, immune checkpoint inhibitors, tyrosine kinase inhibitors, and curative radiotherapy).

**Results:**

Among 204 patients with solid cancer and 73 healthy controls, 65% of SARS-CoV-2-naïve patients with cancer developed anti-S antibodies after the first vaccine dose, rising to 92% after the second dose. By 6 months, all BNT162b2-vaccinated patients with solid cancer developed antibody response. Patients treated with CT showed impaired both humoral and cellular immune response to BNT162b2 vaccination. Antibody levels in SARS-CoV-2-recovered patients were comparable to healthy controls. T-cell response peaked after the second dose of BNT162b2 and was not significantly impaired in solid cancer patients except those treated with CT.

**Conclusion:**

Immune response to BNT162b2 COVID-19 mRNA vaccine is substantially shaped by pre-vaccination COVID-19 infection. All patients with solid cancer on active anticancer therapy exhibited seroconversion after COVID-19 vaccination, although the extent of both humoral and cell immune response was substantially hampered in those treated by CT.

**Trial registration:**

EudraCT No. 2021-000566-14 (registration date February 17, 2021).

## Introduction

According to the latest WHO update from November 2024, more than 776 million cases of COVID-19 have been reported, with more than 7 million reported COVID-19 deaths.^1^ Patients with cancer are among the most vulnerable groups presenting with the most severe clinical course and highest burden of COVID-19 deaths during all waves of the COVID-19 pandemic. To date, vaccination has been the only systemic measure to protect the population from COVID-19 infection.^2–4^

Active systemic treatment of cancer may assumingly weaken the effect of vaccination.^5–7^ Despite the underrepresentation of patients with cancer in vaccine approval trials, early studies evaluating the immune response of COVID-19-vaccinated patients with cancer showed a reduced humoral response after a double dose of BNT162b2 compared to healthy cohorts.^8–10^ Regarding cellular immunity, COVID-19 vaccines induce a CD4^+^ response, with IFN-γ, TNF-α, and IL-2 production observed in both pathogen-specific CD4^+^ and CD8^+^ T. More recent data on cancer patients suggest that patients with solid tumors (ST) and hematologic malignancies also elicited a cellular immune response after completing COVID-19 mRNA vaccines.^11–13^

In the Czech Republic, a national vaccination program for vulnerable groups was implemented in mid-March 2021. At the same time, the academic phase IV CoVigi clinical trial was designed and initiated. The goal of the prospective multicentric clinical trial was to evaluate COVID-19 vaccination-related adverse events, vaccination efficacy, and anti-SARS-CoV-2 humoral (antibody) and cellular (T-cell) immune response in patients with solid cancer, patients with hematologic malignancies, and reference (control population) prime-vaccinated with COVID-19 mRNA vaccine.

Here, we report data focused on humoral and cellular immune responses in a cohort of patients with solid cancer undergoing anticancer treatment and the control group of healthy volunteers. We address the time-course pattern of anti-S antibodies up to 6 months from prime vaccination and the onset of SARS-CoV-2 specific T-cell response after Pfizer-BioNTech vaccine BNT162b2 in the context of previous SARS-CoV-2 infection and anticancer therapy.

## Materials and methods

### Study design and study population

CoVigi is the phase IV prospective open-label non-randomized multicentric clinical study (EudraCT No. 2021-000566-14) evaluating COVID-19 vaccination adverse events, efficacy, and immune response time course in cancer patients on active anticancer therapy and prime-vaccinated against COVID-19.

Two academic hospitals, University Hospital Brno and Masaryk Memorial Cancer Institute, participated in enrolling 500 subjects. Patients with all types of ST, hematologic malignancies (not presented here), and a reference cohort (RC) composed of healthy volunteers mainly recruited from, but not limited to, employees of Masaryk University were invited to enter this study. All participants were 18 years or older with a life expectancy of at least 6 months. Pregnancy, breastfeeding, and prior vaccination against COVID-19 constituted exclusion criteria for the CoVigi trial. COVID-19 vaccines (ATC group J07BX03) according to the national COVID-19 vaccination priorities entering the CoVigi trial were as follows: COMIRNATY Pfizer-BioNTech, COVID-19 Vaccine Moderna, COVID-19 Vaccine VAXZEVRIA, and COVID-19 Vaccine Janssen. The first patient was included in the study on March 22, 2021.

The reporting of this study conforms to the guidelines set forth by Equator Network, following the STROBE statement (Supplemental Material).

A questionnaire survey addressing the onset and course of COVID-19 disease and self-reported vaccination side effects (not presented here) were collected, and immune characteristics, including quantitative analysis of anti-S and anti-N SARS-CoV-2 antibodies and SARS-CoV-2-specific T-cell response assessed by IGRA (IFN-γ-release assay), were evaluated at each study visit and recorded. The clinical trial plan is depicted in Supplemental Figure 1. The study was listed as EudraCT No. 2021-000566-14 (registration date February 17, 2021).

The groups to be analyzed were defined as (i) subjects from the RC without a diagnosis of cancer and (ii) solid cancer patients who underwent anticancer treatment during the COVID-19 vaccination. Anticancer treatment subcohorts were defined: chemotherapy (CT) or chemotherapy + monoclonal antibodies (CT + mAb), monoclonal antibodies (mAb), immune checkpoint inhibitors (ICI), tyrosine kinase inhibitors (TKI), and curative radiotherapy (RT). In addition, subgroups by demographic characteristics were considered. All subjects in this report received 30 mg of the double-dose BNT162b2 vaccine intramuscularly. Patients with other vaccine types were excluded due to the predominance of the COMIRNATY vaccine in the study population.

Blood samples from all study participants were collected to evaluate the anti-SARS-CoV-2 immune response and analyzed here at the following time points: prior to vaccination (V1), prior to the second dose (V2), 28–58 days after the first dose (V3), at 3 months (V4), and 6 months (V5). Data from the following visits (12 months/V6, 18 months/V7, 24 months/V8) were collected but not analyzed here (revaccination period).

### Antibody and SARS-CoV-2-specific T-cell response testing

#### Antibody response

Blood was collected by venipuncture in a Li-heparin tube. Plasma was separated within 4 h from blood collection and tested within the same day. Total anti-N antibodies were evaluated using Elecsys^®^ Anti-SARS-CoV-2 immunoassay and total anti-S antibodies were quantified using Elecsys Anti-SARS-CoV-2 S immunoassay using Roche Cobas e 411 analyzer. Plasma with anti-S above 250 and 25,000 U/ml were diluted 100-fold and 1000-fold, respectively, and reanalyzed. The total anti-N Elecsys Anti-SARS-CoV-2 antibody assay targets nucleocapsid (N) antigen, results are in the form of a cut-off index (COI), with levels ⩾0.5 COI were considered reactive. The total anti-S Elecsys Anti-SARS-CoV-2 assay targets RBD spike protein and detects total anti-SARS-CoV-2 antibodies (IgG, IgA, and IgM). Anti-S antibody levels ⩾0.8 U/ml were considered positive, and the Binding Antibody Units (BAU) conversion factor was 1.288.^14^

#### SARS-CoV-2-specific T-cell response

Blood was collected into plain heparin tubes and incubation with antigens was initiated within 4 h from blood collection as follows: 1 ml of blood was transferred into antigen-specific tubes containing CD4^+^ T-cell epitopes from the S1 subunit of the spike protein (Ag1 tube) and both CD4^+^ and CD8^+^ T-cell epitopes derived from the S1 and S2 subunits of the spike protein (Ag2 tube). The control set tubes include negative (Nil) and positive (mitogen) controls. Nil value was subtracted from Ag1, Ag2, and positive control value, respectively, and the cut-off level for a positive response was 0.15 pg/ml (subtracted). Data from Ag2 tubes targeting CD4^+^ and CD8^+^ T-cell epitopes were analyzed and presented here. All specimens passed positive/mitogen control.

### Statistical analysis

The objectives of the CoVigi trial were exploratory by nature and the sample size was determined pragmatically and appropriately for the study as 500 subjects. The standard methods of descriptive statistics were used, that is, numbers and percentages for categorical characteristics and median and interquartile range for continuous characteristics. Comparison of anti-S antibody and SARS-CoV-2-specific T-cell response levels between subcohorts was performed using non-parametric Mann–Whitney or Kruskal–Wallis tests. Fisher’s exact test was used to compare the positivity of SARS-CoV-2-specific T-cell response between solid cancer patients and RC. All statistical analyses were performed using R version 4.3.1 and a common significance level of 5%.^15^

## Results

### Subject characteristics

**Figure 1.**
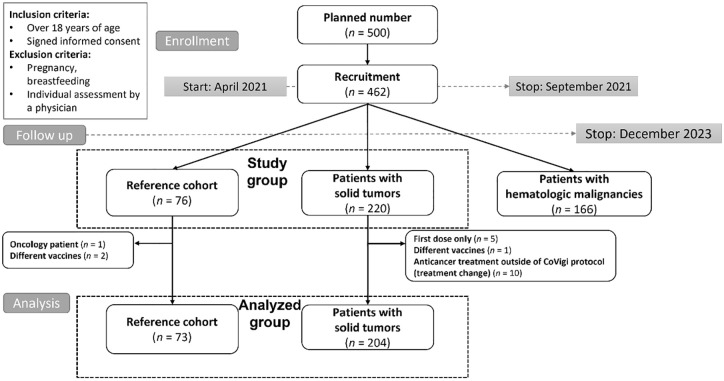
Consort diagram. The analyzed group was represented by (i) subjects from the reference cohort without a diagnosis of cancer and (ii) patients with solid cancer who underwent the anticancer treatment modality defined for this study.

The study population is depicted in Figure 1. Data from the solid tumor cohort (STC; *n* = 204) and RC (*n* = 73) were analyzed.

Subject characteristics are summarized in Table 1. Cancer diagnosis included predominantly breast carcinoma, gastrointestinal, and genitourinary cancers.

**Table 1.** Characteristic of the analyzed subjects by study cohort.

Characteristic	Overall, *N* = 277	Reference cohort, *N* = 73	Solid tumor cohort, *N* = 204
Age (years)			
Median (IQR)	55 (41, 63)	39 (31, 47)	59 (49, 66)
Range	18, 77	18, 65	22, 77
⩽40	62 (22%)	41 (56%)	21 (10%)
41–50	57 (21%)	18 (25%)	39 (19%)
51–60	67 (24%)	10 (14%)	57 (28%)
61–70	71 (26%)	4 (5.5%)	67 (33%)
>70	20 (7.2%)	0 (0%)	20 (9.8%)
Sex			
Female	191 (69%)	52 (71%)	139 (68%)
Male	86 (31%)	21 (29%)	65 (32%)
Last available visit			
V3	6 (2.2%)	0 (0%)	6 (2.9%)
V4	8 (2.9%)	0 (0%)	8 (3.9%)
V5	21 (7.6%)	1 (1.4%)	20 (9.8%)
V6	24 (8.7%)	4 (5.5%)	20 (9.8%)
V7	7 (2.5%)	0 (0%)	7 (3.4%)
V8	211 (76%)	68 (93%)	143 (70%)
Cancer diagnosis			
BC			91 (45%)
GI cancer			45 (22%)
GU cancer			34 (17%)
Melanoma			13 (6.4%)
H&N and lung			15 (7.4%)
Other			6 (2.9%)
Anticancer treatment			
TKI			54 (26%)
mAb			37 (18%)
ICI			20 (9.8%)
CT			61 (30%)
CT + mAb			26 (13%)
RT			6 (2.9%)
ECOG performance status			
0			123 (61%)
1			79 (39%)
2			1 (0.5%)
Unknown			1

BC, breast carcinoma; CT, chemotherapy; ECOG, Eastern Cooperative Oncology Group; GI, gastrointestinal; GU, genitourinary; H&N, head and neck; ICI, checkpoint inhibitors; IQR, interquartile range; mAb, monoclonal antibodies; RT, radiotherapy; TKI, tyrosine kinase inhibitors.

### Pre-vaccination anti-SARS-CoV-2 seropositivity

Pre-vaccination anti-N and anti-S antibody levels were evaluated from the specimens collected at V1 (RC *n* = 75, STC *n* = 210). Anti-N or anti-S antibody reactivity was detected in 29% of STC and 37% of RC prior to the COVID-19 vaccination (Supplemental Figure 2). The subgroup of seropositive pre-vaccination was termed “SARS-CoV-2-recovered”. Subjects with negative anti-SARS-CoV-2 antibody levels pre-vaccination were perceived as “SARS-CoV-2-naïve”. Levels of anti-S antibodies for SARS-CoV-2-recovered subjects did not differ between RC and STC, nor were they influenced by the type of anticancer therapy. A questionnaire (V1) analysis revealed that a substantial proportion of SARS-CoV-2-recovered subjects (44% in the STC and 32% in the RC, respectively) did not self-report COVID-19 disease before the vaccination (Supplemental Figure 2). No seronegative subject reported COVID-19 disease or PCR positivity in pre-vaccination history (Supplemental Figure 2).

### Seroconversion rate, levels, and the time-course pattern of post-vaccination anti-SARS-CoV-2 antibody levels in SARS-CoV-2-naïve and SARS-CoV-2-recovered subjects

The median levels of SARS-CoV-2 anti-S antibodies in individual visits are summarized in Table 2 separately for RC and STC and separately for SARS-CoV-2-naïve and recovered cohorts. In the RC, all SARS-CoV-2-naïve individuals developed antibody responses after the first dose of the vaccine. In SARS-CoV-2-naïve patients with ST, 65% of individuals were positive for anti-S antibodies after the first vaccine dose (V2), 92% exhibited seroconversion within 3 weeks after the second dose of vaccine (V3), and 99% and 100% after the 3 months (V4) and 6 months (V5), respectively, after vaccination (Table 2).

**Table 2.** Seroconversion rate and median levels of anti-S antibody during the COVID-19 vaccination course separately for reference and solid tumor cohorts and SARS-CoV-2-naïve and recovered subjects.

Cohorts	V1, pre-vaccination		V2, after the first dose		V3, after the second dose		V4, 3 months		V5, 6 months	
		*p*-Value^a^		*p*-Value^a^		*p*-Value^a^		*p*-Value^a^		*p*-Value^a^
SARS-CoV-2-naïve										
Reference cohort, *N* = 47										
Median (U/ml)	0.4	NS	93.7	**<0.001**	13,790	**<0.001**	6115	**<0.001**	1526	**<0.001**
Seroconversion	0%		100%		100%		100%		100%	
Solid tumor, *N* = 143										
Median (U/ml)	0.4		2.6		527		397		286	
Seroconversion	0%		65%		92%		99%		100%	
SARS-CoV-2-recovered^b^										
Reference cohort, *N* = 26										
Median (U/ml)	82.7	0.633	43,745	**<0.001**	55,595	**0.030**	14,520	0.350	5400	0.447
Solid tumor, *N* = 61										
Median (U/ml)	120.6		11,100		24,160		11,520		4733	

a *p*-Value refers to the difference in anti-S levels between the reference and solid tumor cohort.

b All SARS-CoV-2 recovered subjects were seropositive for anti-S antibody, seroconversion rate was 100%.

Statistically significant *p*-Values are highlighted in bold.

**Figure 2.**
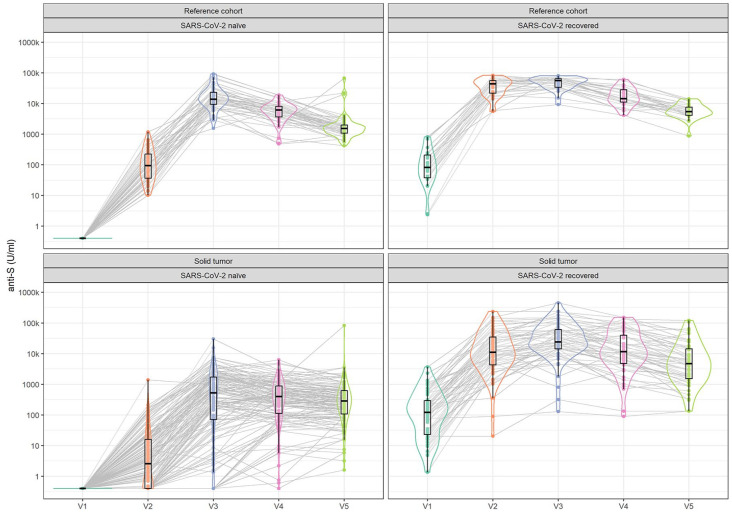
Time-course patterns of pre- and post-vaccination anti-S antibody levels in SARS-CoV-2-naïve and SARS-CoV-2-recovered subjects from reference and solid tumor cohorts. V1 = pre-vaccination, V2 = after first dose, V3 = after second dose, V4 = 3 months, V5 = 6 months.

The levels and the time course of post-vaccination anti-S antibodies were substantially influenced by pre-vaccination SARS-CoV-2 infection (Table 2 and Figure 2). Without previous exposure to the SARS-CoV-2 virus, patients with solid cancer produced substantially lower levels of anti-S antibodies compared to reference individuals at all time points up to 6 months (*p* < 0.001). However, in those infected with SARS-CoV-2 prior to vaccination, differences in anti-S levels between STC and the RC were observed at V2 point (*p* < 0.001) and V3 point (*p* = 0.030) but later diminished at V4 and V5 points after the first dose (*p* = 0.350 and *p* = 0.447 at V4 and V5, respectively).

Levels of anti-N antibodies remained non-reactive in SARS-CoV-2-naïve subjects except for several cases that experienced SARS-CoV-2 infection during follow-up. In SARS-CoV-2 recovered subjects, anti-N antibody reactivity declined except for those re-infected by SARS-CoV-2 during follow-up or shortly before vaccination (Supplemental Figure 3).

### Pre-vaccination and time-course patterns of post-vaccination SARS-CoV-2-specific T-cell response in SARS-CoV-2-naïve and SARS-CoV-2-recovered subjects

In the SARS-CoV-2-naïve subgroup, SARS-CoV-2-specific T-cell response was detected in 2.1% and 0.7% in the RC and STC, respectively. T-cell response positivity peaked after the second dose (V3), with 79% positive subjects in the RC and 73% in the STC. In individuals without previous exposure to the SARS-CoV-2 virus, substantially lower SARS-CoV-2-specific T-cell response was observed in STC compared to RC after the first dose of vaccine (V2, *p* < 0.001). Surprisingly, after the second dose of vaccine (V3), higher SARS-CoV-2-specific T-cell response levels were observed in SARS-CoV-2-naïve subjects with solid cancer at the margin of statistical significance (*p* = 0.092) (Table 3).

**Table 3.** Median levels (IGRA) and positivity of SARS-CoV-2-specific T-cell response during the COVID-19 vaccination course separately for reference and solid tumor cohorts and SARS-CoV-2-recovered and naïve cohorts.

Cohorts	V1, pre-vaccination		V2, after the first dose		V3, after the second dose		V4, 3 months		V5, 6 months	
		*p*-Value		*p*-Value		*p*-Value		*p*-Value		*p*-Value
SARS-CoV-2-naïve										
Reference cohort, *N* = 47										
Median (pg/ml)	0.00	0.590	0.09	**<0.001**	0.46	0.092	0.18	0.787	0.13	0.981
Positivity	2.1%	0.441	45%	**<0.001**	79%	0.401	55%	0.946	47%	0.972
Solid tumor, *N* = 143										
Median (pg/ml)	0.00		0.03		0.63		0.18		0.11	
Positivity	0.7%		16%		73%		55%		47%	
SARS-CoV-2-recovered										
Reference cohort, *N* = 26										
Median (pg/ml)	0.05	0.850	0.70	0.290	1.08	0.078	0.69	0.159	0.40	0.213
Positivity	12%	0.207	85%	0.199	100%	**0.008**	85%	0.272	77%	0.100
Solid tumor, *N* = 61										
Median (pg/ml)	0.04		0.45		0.55		0.36		0.19	
Positivity	23%		72%		78%		74%		58%	

IGRA, IFN-γ-release assay.

*p*-Values refers to the difference between the reference and solid tumor cohort. Statistically significant *p*-Values are highlighted in bold.

**Figure 3.**
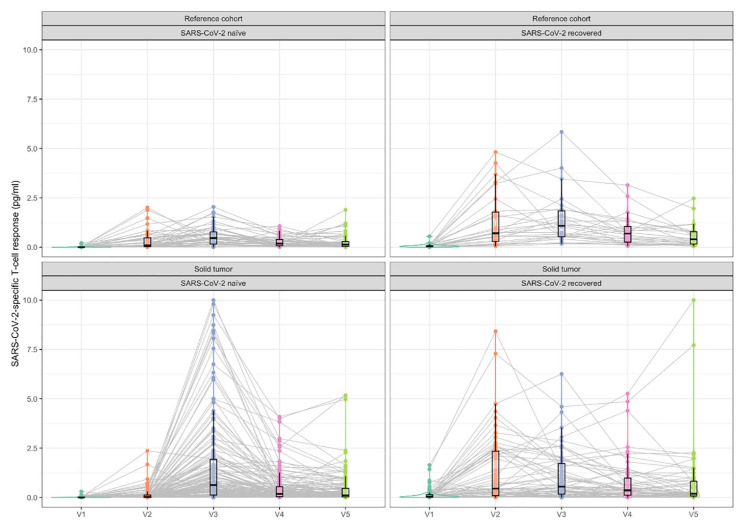
Time-course pattern of post-vaccination SARS-CoV-2-specific T-cell response in SARS-CoV-2-naïve and SARS-CoV-2-recovered separately for reference and solid tumor cohorts.

In the SARS-CoV-2-recovered subgroup, SARS-CoV-2-specific T-cell response was detected in 12% and 23% in RC and STC, respectively, and the positivity rate after the first dose was substantially higher compared to those SARS-CoV-2 naïve. Again, T-cell response positivity peaked after the second dose (V3) with 100% and 78% for RC and STC, respectively, and remained positive in the majority of SARS-CoV-2-recovered subjects up to 6 months from vaccination. Here, the reference subjects had slightly better SARS-CoV-2-specific T-cell response. Particularly, the prevalence was 100% in the RC after the second dose of vaccine (V3) compared to 78% in the STC (*p* = 0.008; Table 3 and Figure 3).

To address the possible presence of SARS-CoV-2-specific T-cell response in seronegative individuals, the simultaneous presence and absence of humoral and cell immune response were analyzed (Supplemental Table 1). SARS-CoV-2-specific T-cell positivity in the IGRA test was observed in 2.1% of seronegative individuals from the RC prior to vaccination. In seronegative STC, SARS-CoV-2-specific T-cell response was observed in 0.7% (prior to vaccination), 2.1% (after the first dose), and 3.5% (after the second dose). The rates of specific T-cell response in seronegative individuals are very low on the level of background noise caused by analytical uncertainty. However, a possible contribution of T-cell cross-reactivity to common coronaviruses cannot be excluded at this time.

### Effect of age and sex on post-vaccination anti-SARS-CoV-2 antibody level and virus-specific T-cell response

The effect of sex and age on anti-S antibody level was evaluated in the SARS-CoV-2-naïve subgroup of the reference population (*n* = 47) representing a homogeneous group affected just by COVID-19 vaccination. Regarding sex, levels of anti-S post-vaccination antibodies did not differ between men and women at any time point investigated (Supplemental Figure 4). Regarding T-cell response and sex, a higher level of T-cell response was observed in women compared to men after the first dose of the vaccine and 6 months after vaccination (Supplemental Figure 5).

Regarding age, no association between age and post-vaccination antibody level or SARS-CoV-2 specific T-cell response was observed (Supplemental Figures 6 and 7).

### Effect of ongoing anticancer therapy type on immune response to COVID-19 vaccination

**Figure 4.**
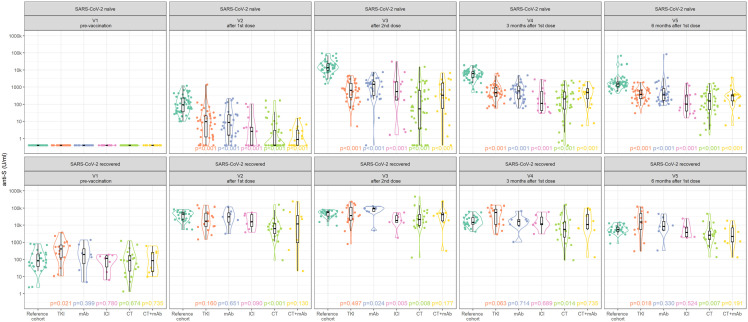
Time-course patterns of post-vaccination anti-S antibody levels in SARS-CoV-2-naïve and SARS-CoV-2-recovered patients with solid tumors by anticancer therapy.

In SARS-CoV-2-naïve subjects, a substantial proportion (35%) of patients with solid cancer remained seronegative after the first dose of vaccine (V2), showing differences related to anticancer therapeutic modalities, specifically TKI 16%, mAb 13%, ICI 46%, CT 62%, and CT + mAb 44% seronegative. After the second dose of the vaccine (V4), 8% of patients with solid cancer remained seronegative (mAb 3%, CT 21%, CT + mAb 6%, and 0% for TKI and ICI). Three months after the first dose (V4), only one patient treated with CT (3%) remained seronegative, and all patients with solid cancer showed seroconversion by month 6 after the vaccination (V5). At all time points, all anticancer therapy subcohorts had significantly lower anti-S antibody levels compared to the RC for SARS-CoV-2-naïve subjects (*p* < 0.001). In SARS-CoV-2-recovered subjects, lower levels of anti-S antibodies compared to the RC were observed mainly in patients with solid cancer treated with CT (*p* < 0.001 at V2, *p* = 0.008 at V3, *p* = 0.014 at V4, *p* = 0.007 at V5). Other anticancer therapy modalities exhibited similar or higher levels of anti-S compared to the RC. The time-course pattern of post-vaccination anti-S antibody levels by anticancer therapy modality is shown in Figure 4.

**Figure 5.**
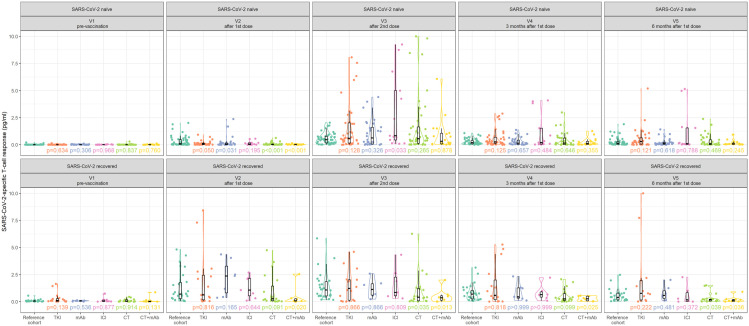
Time-course pattern of post-vaccination SARS-CoV-2-specific T-cell response in SARS-CoV-2-naïve and SARS-CoV-2-recovered patients with solid cancer grouped by anticancer therapy.

Regarding SARS-CoV-2-specific T-cell response, comparable prevalence was observed for all anticancer subcohorts as for the RC with the only exception of CT and CT + mAb subcohorts. Importantly, in SARS-CoV-2-naïve subjects after the first dose of the vaccine, SARS-CoV-2-specific T-cell response was observed in 45% of reference individuals and 10% and 0% of solid cancer patients treated with CT and CT + mAb, respectively. At subsequent visits (V3–V5), the virus-specific T-cell response rate was at the levels of our RC. The time-course patterns of post-vaccination SARS-CoV-2-specific T-cell response in SARS-CoV-2-naïve and SARS-CoV-2-recovered patients with solid cancer are shown grouped by anticancer therapy in Figure 5.

## Discussion

This study presents unique longitudinal data on the humoral and cellular immune responses in patients with ST who were vaccinated with the BNT162b2 COVID-19 vaccine and in healthy volunteers. The data were collected following the initial administration of the BNT162b2 COVID-19 vaccine as part of a prospective multicentric phase IV clinical trial. Substantial qualitative and quantitative differences were identified in SARS-CoV-2-specific immune response shaped by infection with SARS-CoV-2 virus prior to the vaccination. As expected, striking differences between recovered and naïve cohorts were observed after the first dose of the vaccine. After the second dose of COVID-19 vaccination, the augmenting effect of SARS-CoV-2 infection on post-vaccination antibody levels became less pronounced in RC. On the other hand, post-vaccination antibody levels in SARS-CoV-2-recovered patients with solid cancer remained substantially higher compared to SARS-CoV-2-naïve patients with solid cancer up to 6 months from the vaccination. As a result, SARS-CoV-2-recovered vaccinated patients with solid cancer had comparably high levels of anti-S antibodies as the reference population after infection and vaccination. A similar pattern was observed for virus-specific T-cell response, that is, SARS-CoV-2-recovered individuals developed faster, and stronger immune responses than SARS-CoV-2-naïve individuals. Interestingly, SARS-CoV-2-specific T-cell response in patients with cancer was not inferior to the RC.

In SARS-CoV-2-recovered individuals, the level of naturally developed anti-S antibodies pre-vaccination was not inferior in STC compared to the RC. Although we observed quantitatively lower levels of anti-S antibodies in SARS-CoV-2-naïve patients with cancer, 92% of patients were seroconverted within 3 weeks after the second dose of vaccine and 98% and 100% after 3 and 6 months, respectively. Our results are consistent with previously published systematic reviews.^16–18^ These reviews showed that despite seroconversion rates being significantly lower in immunocompromised patients, the second dose of vaccination was associated with improved seroconversion even in patients with solid cancer. The largest systematic review and meta-analysis on naïve SARS-CoV-2 patients was published recently.^13^ In this meta-analysis, 92% of patients with solid cancer were seroconverted 2 months after the baseline vaccination. The overall rate of cellular response since vaccination was 68%. These findings are consistent with the results of our study that T-cell positivity peaked after the second dose (V3), with 73% of patients with ST testing positive.

Regarding the immune response dynamics, antibody and T-cell response peaked after the second dose of the COVID-19 vaccine and gradually declined. Antibody levels, however, remained reactive up to 6 months after vaccination in all analyzed subjects. This finding aligns with a similar dataset of patients with solid cancer (*n* = 201) who experienced a decrease from 93% to 86% 3 months after vaccination.^12^

In our study, the SARS-CoV-2-specific T-cell response rate declined, and the virus-specific T cells were undetectable in a substantial proportion of individuals (approximately 1/2 and 1/3 in SARS-CoV-2 naïve and recovered, resp.). However, SARS-CoV-2-specific T cells are not confined to the bloodstream, and the lack of their presence in the peripheral blood (revealed by IGRA) does not exclude the presence of SARS-CoV-2-specific memory T cells in secondary lymphoid organs. The immune response was analyzed based on treatment type to evaluate the distinct immunosuppressive potential of each cancer therapy. Although 62% of patients in the SARS-CoV-2-naïve cancer cohort remained seronegative after the first dose of the vaccine, a humoral response was induced in almost all patients with cancer after the second dose of BNT162b2. According to the anticancer therapy modality, CT-treated patients exhibited weaker antibody responses compared to other therapy modalities analyzed. Rapid seroconversion was achieved in patients treated with mAb alone (13% negativity after the first dose) and, surprisingly, in the TKI subgroup (16% negativity after the first dose). This finding is consistent with published data and provides evidence that other active treatment modalities besides CT did not substantially compromise the humoral immune response.^19,20^ By contrast, Lasagna et al. reported on the long-term follow-up of a small study focused on cancer patients treated with immune ICI, with or without CT.^21^ They observed a significant decline in both humoral and cellular responses 6 months after the initial vaccination and advocated for the need for a booster in this patient population.

However, in 2021, a Dutch prospective multicenter study demonstrated the non-inferiority of antibody response after mRNA vaccination in the control group compared to patients treated with immunotherapy alone, CT, or chemoimmunotherapy.^22^

As for T-cell response, we observed a low positivity rate in cancer patients treated with CT compared to other anticancer treatment modalities exhibiting similar SARS-CoV-2-specific T-cell response levels as the RC. Ehmsen et al. demonstrated that only 46% of solid cancer patients elicited T-cell response.^12^ Another dataset presented discordant T-cell responses from humoral responses including the presence of T-cell response in seronegative cases depending on mRNA vaccine type.^23^ Our study had the advantage of a thorough prospective design; all subjects were vaccinated according to the protocol with the BNT162b2 vaccine, and all solid cancer patients were receiving anticancer therapy at the time of vaccination. Thus, it can be reliably stated that most anticancer modalities impair neither humoral nor cellular immune response except CT. The study aimed to include a diverse, real-world population of cancer patients. However, this broad approach led to a limitation: it lacked sufficient power to conclude specific subgroups, such as those with different tumor types or disease stages. In addition, it was not adequately powered to assess the effects of various treatments. Furthermore, we did not record any comorbidities. We also did not analyze the efficacy of the booster vaccine on antibody levels and T-cell response. Finally, the significant difference between the age of the reference and cancer cohorts should be noted; the RC was significantly younger, with a median age of 39 versus 59. Nevertheless, we did not observe any age-related decline in the quality of humoral or cell immune response to COVID-19 vaccination in our dataset.

## Conclusion

Patients with solid cancer who recovered from COVID-19 and BNT162b2 vaccinated had significantly higher levels of anti-S antibodies than their counterparts not infected by SARS-CoV-2, regardless of anticancer treatment. Infection-induced augmentation of antibody response was evident up to 6 months after prime vaccination. Our prospective data showed that solid cancer patients treated with active anticancer therapy developed a humoral and cellular immune response to BNT162b2 vaccination that was impaired in those treated with CT but not with other anticancer therapy modalities (TKI, mAb, and ICI). Even though the primary pandemic has been contained, cancer patients still face significant risks from COVID-19 and other infectious diseases. These findings could help shape and sustain vaccination strategies for solid cancer patients undergoing specific anticancer treatments. They also motivate both physicians and cancer patients to consider vaccination.

## Supplemental Material

sj-docx-1-tam-10.1177_17588359251316224 – Supplemental material for Patterns of SARS-CoV-2-specific humoral and cellular immune response in actively treated patients with solid cancer following prime BNT162b2 COVID-19 vaccination: results from phase IV CoVigi trial Supplemental material, sj-docx-1-tam-10.1177_17588359251316224 for Patterns of SARS-CoV-2-specific humoral and cellular immune response in actively treated patients with solid cancer following prime BNT162b2 COVID-19 vaccination: results from phase IV CoVigi trial by Radka Lordick Obermannova, Iveta Selingerova, Regina Demlova, Dominika Okruhlicova, Jiri Nevrlka, Katerina Cerna-Pilatova, Kristina Greplova, Zdenka Cermakova, Dalibor Valik, Igor Kiss, Marketa Palacova, Alexandr Poprach, Hana Lejdarova, Sarka Selvekerova, Martina Vaneckova and Lenka Zdrazilova-Dubska in Therapeutic Advances in Medical Oncology

sj-docx-2-tam-10.1177_17588359251316224 – Supplemental material for Patterns of SARS-CoV-2-specific humoral and cellular immune response in actively treated patients with solid cancer following prime BNT162b2 COVID-19 vaccination: results from phase IV CoVigi trial Supplemental material, sj-docx-2-tam-10.1177_17588359251316224 for Patterns of SARS-CoV-2-specific humoral and cellular immune response in actively treated patients with solid cancer following prime BNT162b2 COVID-19 vaccination: results from phase IV CoVigi trial by Radka Lordick Obermannova, Iveta Selingerova, Regina Demlova, Dominika Okruhlicova, Jiri Nevrlka, Katerina Cerna-Pilatova, Kristina Greplova, Zdenka Cermakova, Dalibor Valik, Igor Kiss, Marketa Palacova, Alexandr Poprach, Hana Lejdarova, Sarka Selvekerova, Martina Vaneckova and Lenka Zdrazilova-Dubska in Therapeutic Advances in Medical Oncology

## Declarations

**Ethics approval and consent to participate:** The study was approved by the Ethics Committees of Masaryk Memorial Cancer Institute (approval No. 2021/732/MOU) and University Hospital Brno (approval No. 28/21MEK) and was implemented in accordance with Good Clinical Practice and the Declaration of Helsinki (ICH GCP E6(R2)). Written informed consent was received from each patient prior to enrollment.

**Consent for publication:** Not applicable.

**Availability of data and materials:** The data that support the findings of this study are available from the corresponding author upon reasonable request.
