# Supplementary material for: Patterns of SARS-CoV-2-specific humoral and cellular immune response in actively treated patients with solid cancer following prime BNT162b2 COVID-19 vaccination: results from phase IV CoVigi trial
Source: Ther Adv Med Oncol. 2025 May 17;17:17588359251316224. doi: 10.1177/17588359251316224 (PMC12085753; doi:10.1177/17588359251316224)
Supplement: sj-docx-1-tam-10.1177_17588359251316224 – Supplemental material for Patterns of SARS-CoV-2-specific humoral and cellular immune response in actively treated patients with solid cancer following prime BNT162b2 COVID-19 vaccination: results from phase IV CoVigi trial [file sj-docx-1-tam-10.1177_17588359251316224.docx]

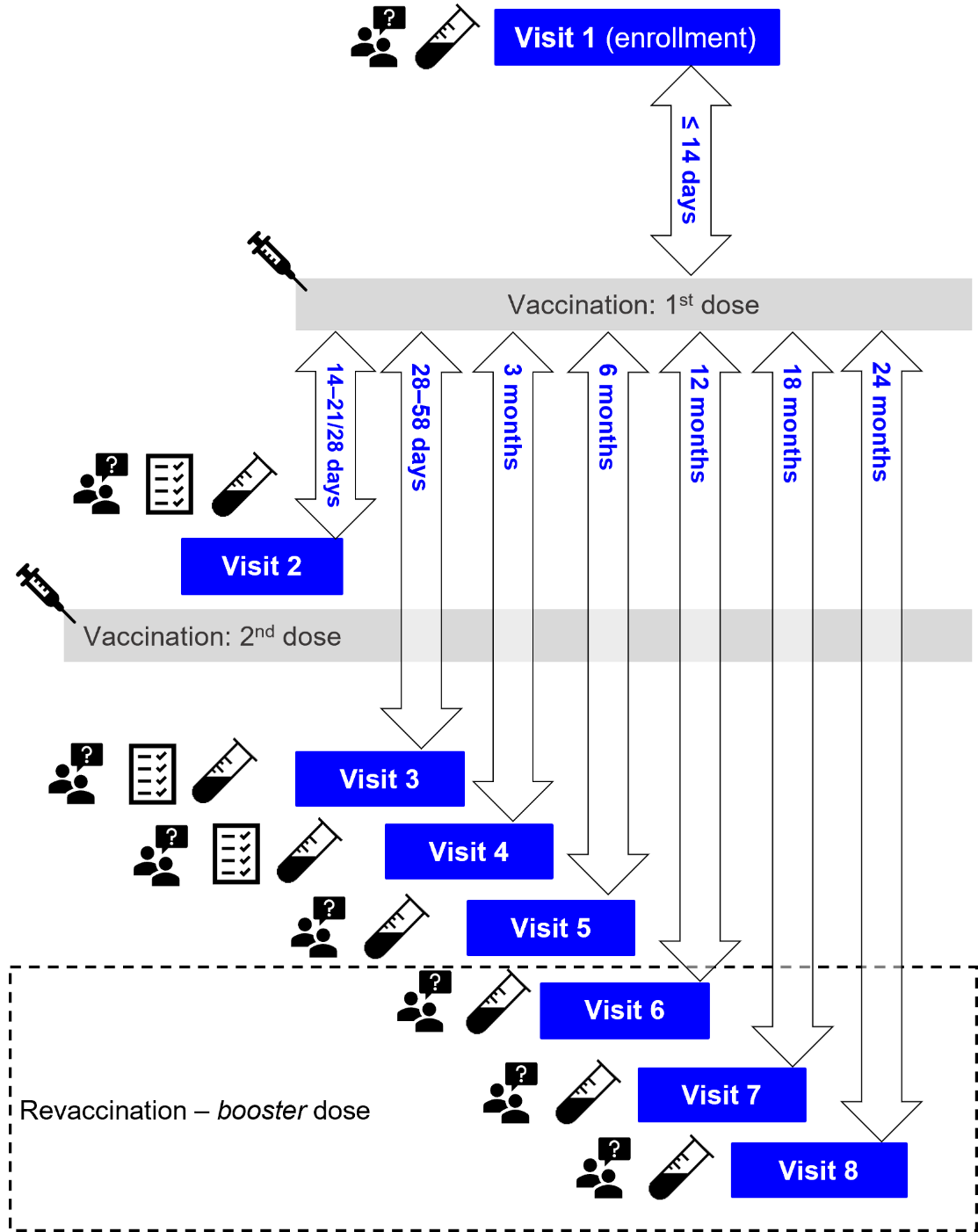


Supplementary Figure 1. Diagram of the scheduled CoVigi trial visits. Visit 1 (V1) -Visit 5 (V5) were analyzed in the manuscript.


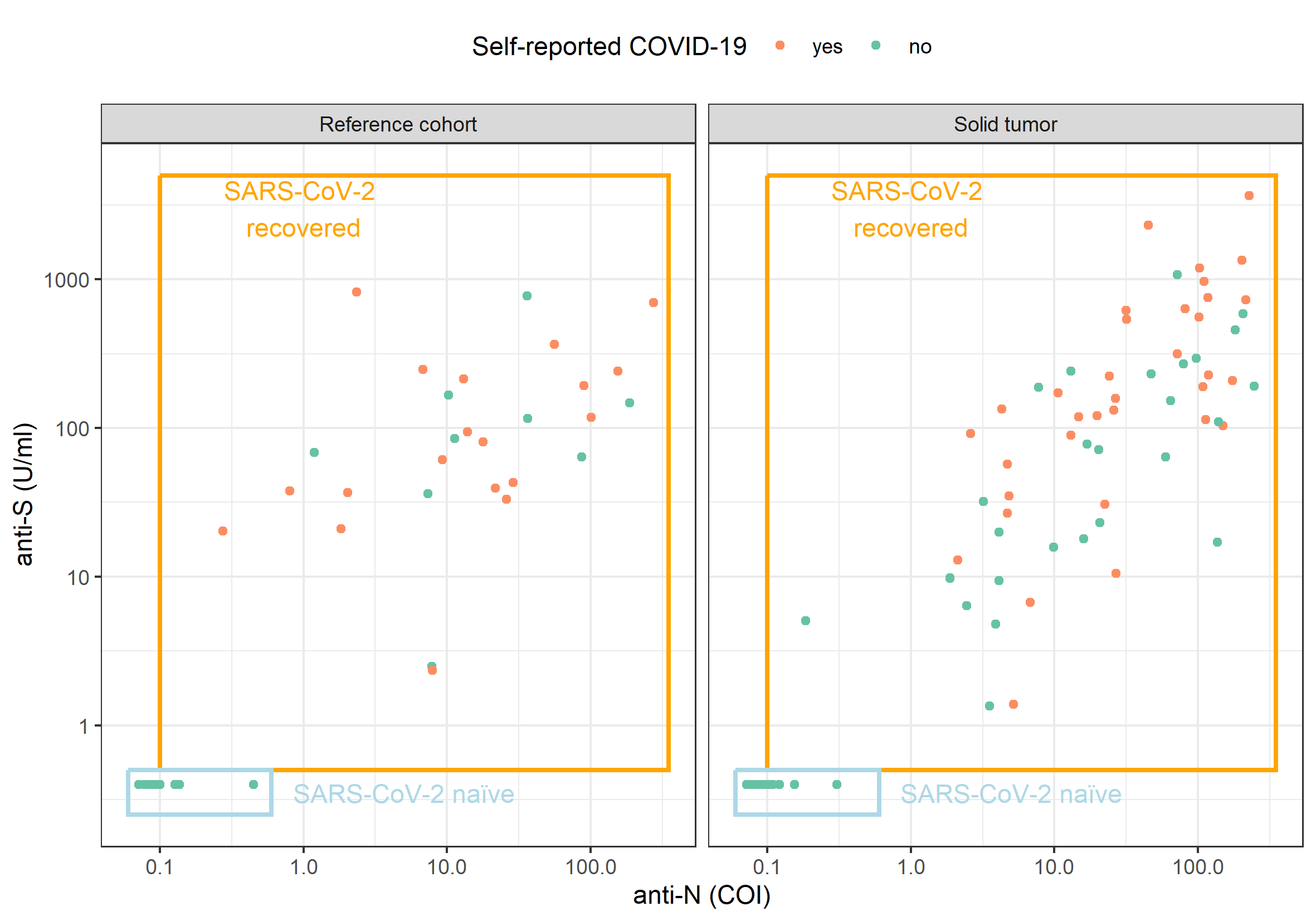


Supplementary Figure 2: The anti-SARS-CoV-2 antibodies pre-vaccination levels in the context of self-reported COVID-19 disease in pre-vaccination medical history.


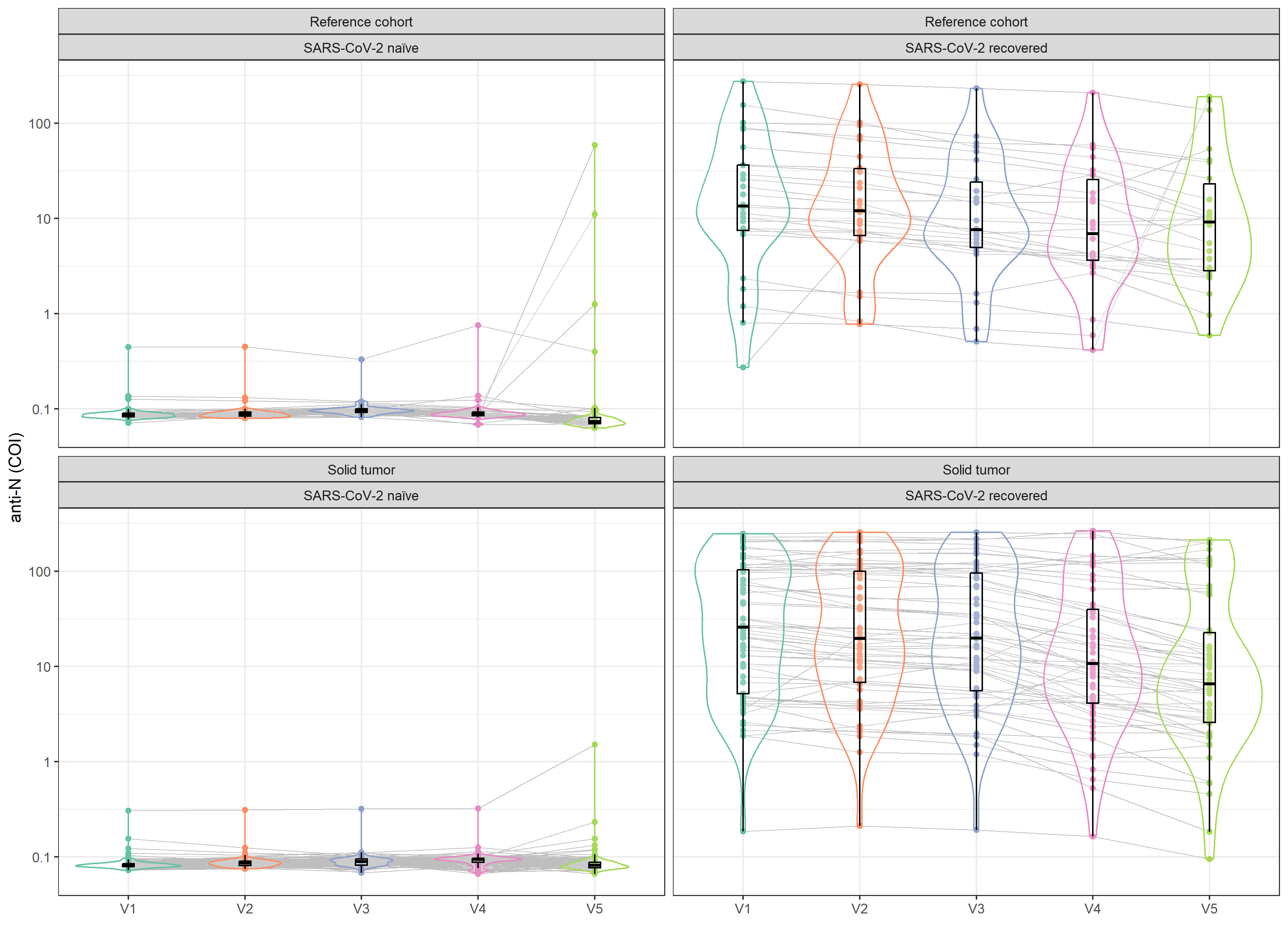


Supplementary Figure 3: Time-course pattern of post-vaccination anti-N antibody reactivity in SARS-CoV-2-naïve and SARS-CoV-2-recovered subjects from reference and solid tumor cohorts


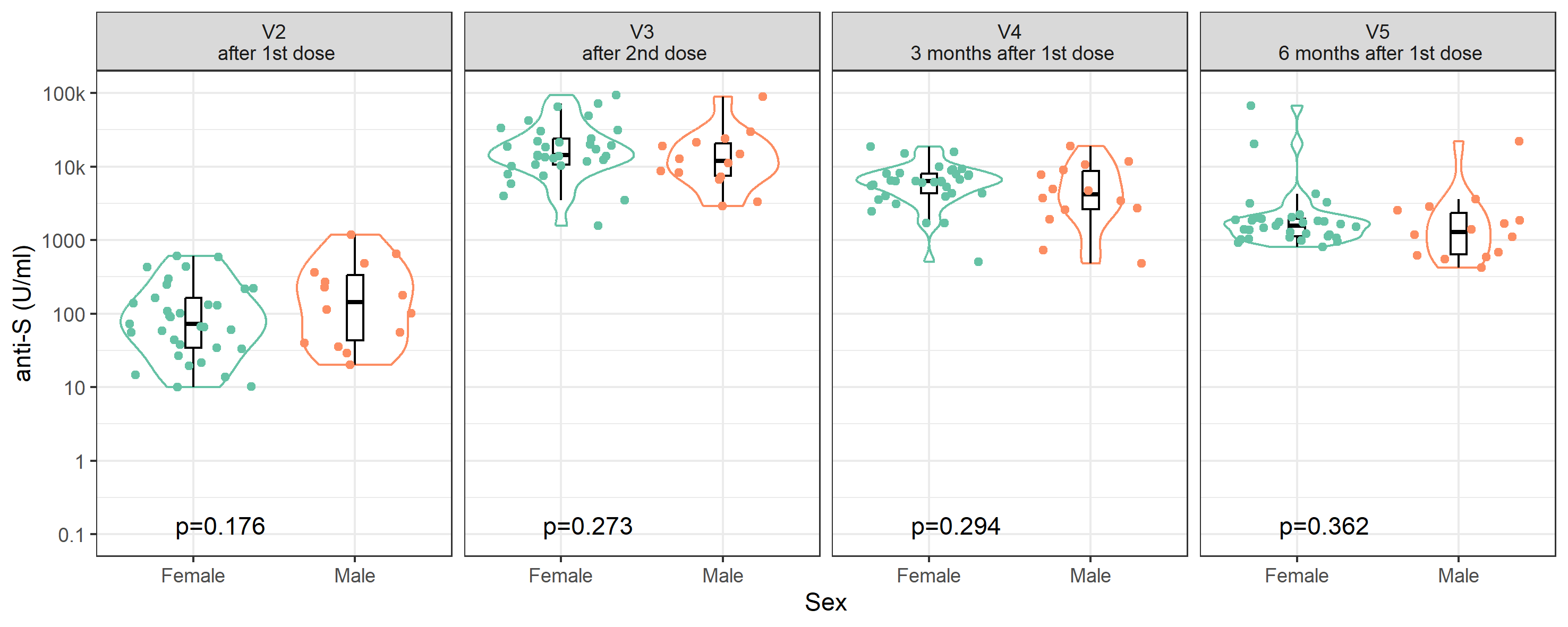
Supplementary Figure 4. Post-vaccination anti-S antibody level in the context of gender in the SARS-CoV-2-naïve subgroup of the reference subcohort.


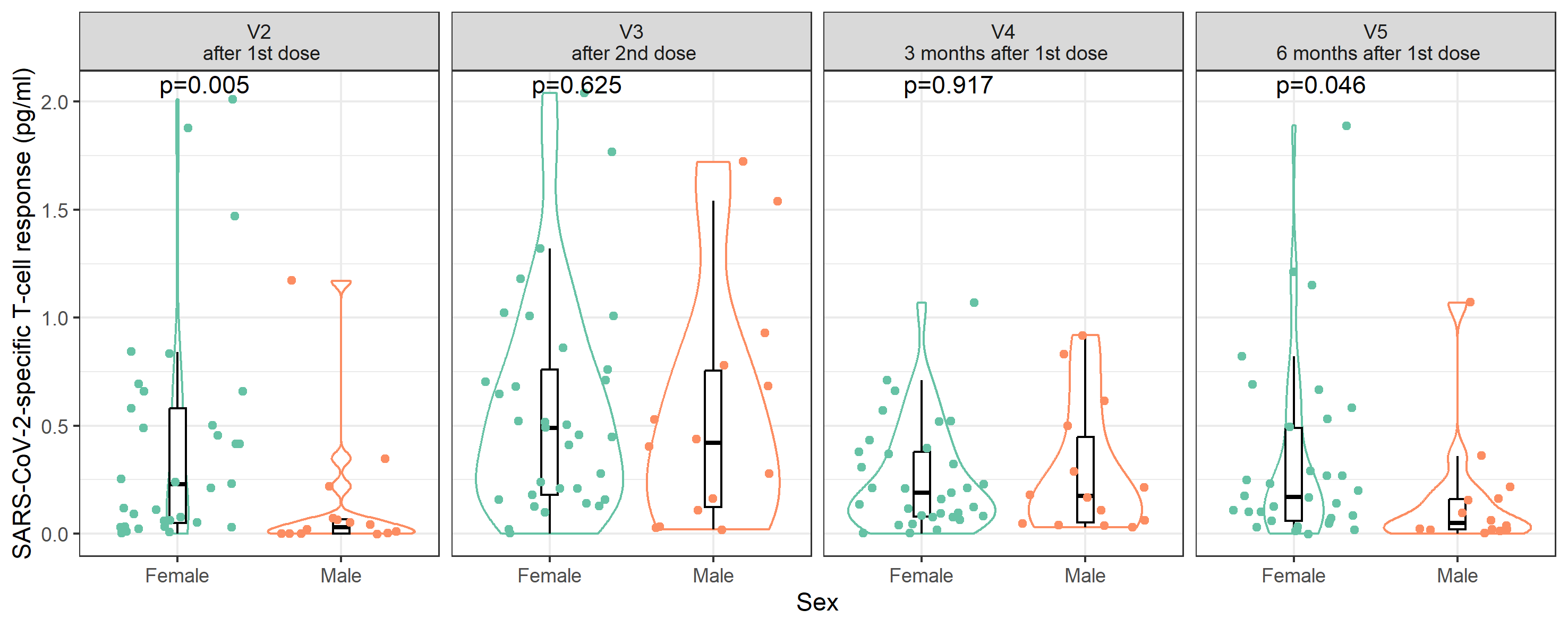
Supplementary Figure 5. Post-vaccination SARS-CoV-2 specific T-cell response in the context of gender in the SARS-CoV-2-naïve subgroup of the reference subcohort.


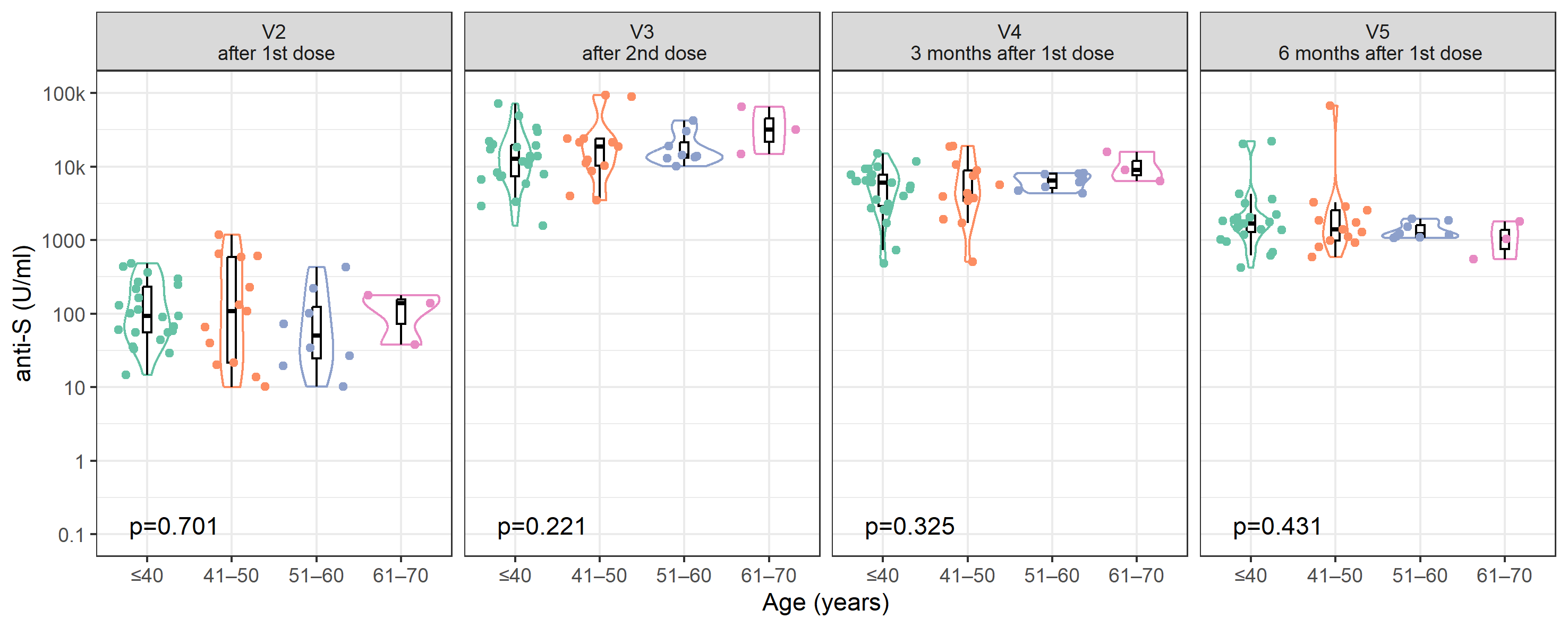


Supplementary Figure 6. Post-vaccination anti-S antibody level in the context of age in the SARS-CoV-2-naïve subgroup of the reference subcohort.


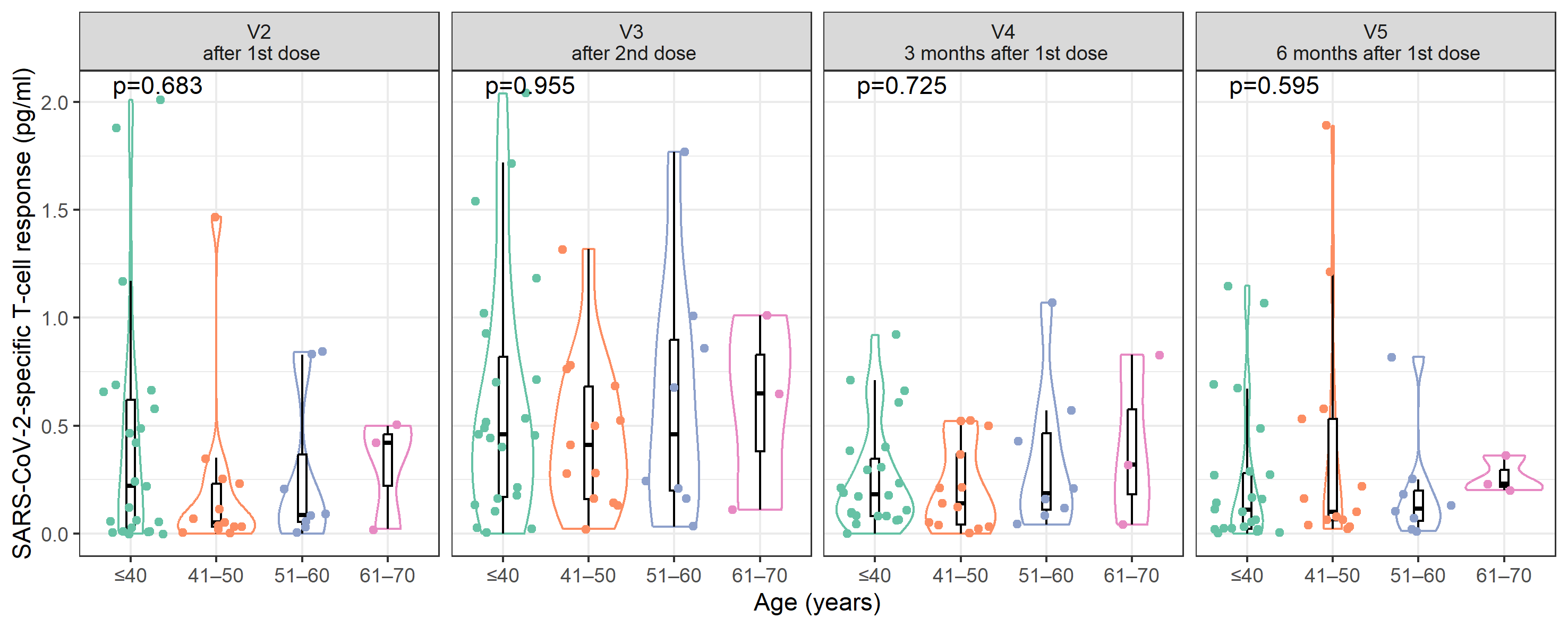
Supplementary Figure 7. Post-vaccination SARS-CoV-2 specific T-cell response in the context of age in the SARS-CoV-2-naïve subgroup of the reference subcohort.

Supplementary Table 1: Post-vaccination association of anti-SARS-CoV-2 antibody and SARS-CoV-2-specific T-cell response in SARS-CoV-2 naïve

|  | **Seropositive anti-S and T-cell response** | | **T-cell response only** | | **Seropositive anti-S only** | | **No immune response** | |
| --- | --- | --- | --- | --- | --- | --- | --- | --- |
|  | **Reference cohort** | **Solid tumor** | **Reference cohort** | **Solid tumor** | **Reference cohort** | **Solid tumor** | **Reference cohort** | **Solid tumor** |
| **V1** | 0% | 0% | 2.1% | 0.7% | 0% | 0% | 98% | 99% |
| **V2** | 45% | 14% | 0% | 2.1% | 55% | 51% | 0% | 33% |
| **V3** | 79% | 69% | 0% | 3.5% | 21% | 23% | 0% | 4.2% |
| **V4** | 55% | 55% | 0% | 0% | 45% | 45% | 0% | 0.7% |
| **V5** | 47% | 47% | 0% | 0% | 53% | 53% | 0% | 0% |
